# Application of Konjac Glucomannan with Chitosan Coating in Yellow Alkaline Noodles

**DOI:** 10.3390/foods12193569

**Published:** 2023-09-26

**Authors:** Shishuai Wang, Jiaxin He, Shanshan Huang, Bin Li

**Affiliations:** 1 College of Food Science and Technology, Wuhan Business University, Wuhan 430056, China; 2 College of Food Science and Technology, Huazhong Agricultural University, Wuhan 430070, China

**Keywords:** chitosan, konjac glucomannan, texture, total plate count, yellow alkaline noodles

## Abstract

To improve the quality of the characteristics of yellow alkaline noodles and enrich their nutritional value, konjac glucomannan (KGM) with or without chitosan coating were added to noodles, and their application effects were investigated in terms of color, texture, water absorption, starch digestion, total plate count (TPC) and microstructure. Chitosan–konjac glucomannan (CK) complex was firstly prepared by embedding konjac powder with chitosan sol. After embedding, the hydrophilicity of KGM decreased significantly. Then, either CK or native KGM were mixed evenly with flour before saline water, and soda was subsequently added to produce noodles. Compared with native KGM, CK provided the noodles with a higher brightness and a lighter yellow color. In terms of texture properties, although the firmness of CK noodles was weaker than that of KGM noodles, the tensile properties were enhanced. After embedding, the water absorption of CK noodles decreased and the content of resistant starch (RS) in the noodles increased. During storage, the TPC in CK noodles was significantly lower than that in KGM noodles. At a CK content of 5%, the noodles presented a lightness of 87.41, a b value of 17.75, a shear work of 39.9 g·cm, a tensile distance of 84.28 cm, a water absorption of 69.48%, a RS content of 17.97% and a TPC of 2.74 lg CFU/g at 10 days. In general, KGM with chitosan coating could improve the physicochemical qualities of noodles and extend their shelf life to a certain extent.

## 1. Introduction

Yellow alkaline noodles (YANs) are very popular in Asian countries, and the consumption of wheat flour in these countries accounts for approximately 30% [1]. It presents a clean yellow surface with a firm and chewy texture [1,2]. At present, more attention is being given to further improve the palatability and nutritional values of YANs. It is a common way to supplement dietary fiber in noodles [3,4,5]. 

As a native water-soluble dietary fiber, konjac glucomannan (KGM), derived from the tuber of konjac, has the advantages of lowering cholesterol, controlling blood sugar levels and promoting digestion [6]. KGM comprises mannose and glucose in a ratio of 1:1.6 or 1:1.4, linked by β-1, 4-glucoside bonds. Its feature of having an acetyl group is randomly distributed in the molecular chain [7]. It was reported that KGM improved the stability, functionality and water retention of wheat gluten by changing the water retention, secondary structure, free sulfhydryl group and disulfide bond contents [8]. The addition of KGM to surimi dough enhanced the starch–gluten–surimi network, and improved the cooking quality and texture of the noodles. It had a major impact on optimizing the macro- and micro-characteristics of dough and noodles [9]. KGM limited the mobility of bound water and immobile water, which hindered starch retrogradation in the noodles [10]. 

The above research showed that KGM could improve the quality of noodles, and the amount of KGM had a significant effect on the texture. Yu et al. [11] found that, compared with other dietary fiber components, KGM had a greater impact on the hardness of cooked noodles than green banana flour. Park et al. [12] pointed out that with the increased content of KGM, the hardness of noodles decreased from 2726 g to 1850 g. He et al. [13] stated that the hardness of steamed bread first declined and then rose with the increasing substitution of KGM. At a substitution level of 5% KGM, the TPA parameters changed dramatically. Scanning electron microscopy proved that the addition of KGM affected the grain structure of cooked starch and the development of the gluten network [14]. In general, sufficient dietary fiber could be appended to improve the nutrition of noodles, but it would result in undesirable negative effects on the texture of noodles. 

Chitosan is a natural cationic polysaccharide extracted from crustaceans such as shrimp and crab. Due to its antibacterial, antioxidant and biodegradation qualities, chitosan is applied in food processing, food ingredients, food preservation, etc. [15,16]. Zhang et al. [17] found that 0.25–2% chitosan greatly enhanced the structure, water holding capacity and antibacterial activity of purple highland barley noodles. Chen et al. [18] investigated the remarkable preservation effect of chitosan oligosaccharides on fresh and wet noodles. Chitosan oligosaccharides could prolong the shelf life of fresh and wet noodles for 3–6 days at 4 °C, and effectively inhibited the growth of acidity value. Tantala et al. [19] suggested that the combination of 0.1% chitosan and −0.1% potassium sorbate could inhibit the growth of mold and microorganisms in fresh noodles at 30 °C for more than 28 days, and showed no adverse effect on the physical properties of noodles, including hardness, whiteness and water activity. 

In view of a favorable bacteriostasis of chitosan in noodles, we attempted to adopt KGM be embedded into chitosan to form the chitosan-coated konjac glucomannan (CK) complex. Whether CK could extend the storage period of noodles was one of our concerns. Furthermore, the previous study found that CK could significantly reduce the hydrophilicity of KGM and avoid the inconvenience caused by the high viscosity of KGM in food processing [20]. Herein, whether it could reduce the excessive swelling of noodles caused by water absorption by KGM during the cooking process was another concern. 

Based on the above hypothesis, CK and native KGM were added to YANs, respectively, and their performance on noodles were compared from the following aspects, such as color, texture, digestive properties, cooking properties and microbial growth. This study can furnish the application of CK in noodle products and aid in the development of functionally healthier noodle products with acceptable qualities. 

## 2. Materials and Methods

### 2.1. Materials

The commercial wheat flour was procured from Wudeli Group Zhoukou Flour Co., Ltd. (zhoukou, China). The contents of moisture, ash, crude protein and wet gluten were 11.95 g/100 g, 0.69 g/100 g, 8.73 g/100 g and 19.58 g/100 g (dry basis, *w*/*w*), respectively. Native KGM powder (purity ≥ 90%; viscosity: 30,000 mPa·s) was kindly provided by Qiang Sheng Co. Ltd. (Jingmen, China). Chitosan (viscosity: 200 mPa·s; deacetylation degree: 90%) was offered by Sinopharm Group Chemical Reagent Co., Ltd. (Beijing, China). D-glucose test kit was from Megazyme Brand and made in Ireland. Pancreatic enzymes and saccharified enzymes were obtained from Shanghai Yuanye Biotechnology Co., Ltd. (Shanghai, China). All the chemicals and reagents used were of analytical grade.

### 2.2. Preparation of Chitosan-Coated Konjac Glucomannan (CK) Powder

CK powder was prepared according to the method of Zhao [20]. First, chitosan was fully dissolved in acetic acid solution containing 10% (*v*/*v*) ethanol. Then, chitosan sol was slowly added to KGM powder and mixed evenly. After elution and neutraliziation with 45% (*v*/*v*) ethanol containing sodium carbonate, the mixture was rinsed with 75% (*v*/*v*) and 100% (*v*/*v*) ethanol. Finally, the mixture was vacuum-dried at 80 °C for 1 h. Thus, chitosan sol was successfully coated on KGM powder to obtain CK powder.

### 2.3. Preparation of Yellow Alkaline Noodles (YANs)

YANs were prepared according to the method of Fan [21]. The basic ingredients of YANs were: 100 g flour, 35 g water, 0.6 g soda and 0.8 g salt. The flour was partially replaced with native konjac powder or CK powder, and other ingredients in YANs remained the same. Based on the weight of dry flour, the mass fraction of native konjac powder or CK powder was set as 1%, 3% and 5%, respectively. The composition of the noodles is shown in Table 1.

### 2.4. Color 

Refer to the method of Koh et al. [22]. Tristimulus values of L (lightness), a (red–green axis) and b (yellow–blue axis) parameters from the noodles were evaluated on a colorimeter (UltraScan XE HunterLab, Reston, VA, USA). Before measurement, the colorimeter was standardized with white and black standard tiles.

### 2.5. Texture

Refer to the method of Yu et al.—with a small modification [11]. The firmness of noodles was measured via the shear mode of a TA-XT plus Texture Analyzer (Stable Microsystems Ltd., Surrey, UK). The probe was selected as A/LKB-F. The pre-test speed, test speed and post-test speed were 1 mm/s, 2 mm/s and 2 mm/s, respectively. The distance was 0.5 mm and the load cell was 5 kg. 

The tensile strength of noodles was determined by the tensile mode. The noodles were fixed on the probe of A/SPR. The initial spacing between probes was controlled to be 15 mm. The speed before, during and after the test were set as 2 mm/s, 1 mm/s and 10 mm/s, respectively.

### 2.6. Water Absorption

Refer to the method of Zhang et al. [23]. After the noodles were cooked in boiling water for different periods of time, water was removed from the surface of the noodles with filter paper, and the mass of noodles was recorded as m_t_. Before cooking, the mass of noodles was noted as m_0_. Water absorption rate of noodles was calculated using the formula:

Water absorption rate (%) = (m_t_ − m_0_)/m_0_ × 100% (1)

### 2.7. Low-Field Nuclear Magnetic Resonance (LF-NMR)

Refer to the method of Ge et al.—with a small modification [10]. The water distribution of semi-dried noodles was determined via a nuclear magnetic resonance imaging analyzer (MesoQMR23-060H, Niumag Electric Corporation, Shanghai, China). About 7 g of samples of about 3 cm in length were placed in the NMR tube and sealed with cling film to prevent moisture evaporation. Parameters measured were SW of 200 kHz, TW of 100 ms, TD of 24022, NS of 8 and NECH of 600.

### 2.8. In Vitro Digestion Properties

Refer to the method of Sun et al.—with a small modification [24]. Noodles were cut into pieces, added with 10 mL enzyme solution and 10 mL buffer solution (pH5.3), and stirred thoroughly at 37 °C. At various time points (0 min, 20 min and 120 min), 2 mL of the mixture was taken out and immediately placed in boiling water to stop the reaction. After cooling and centrifugation, 0.1 mL supernatant was collected, mixed with 3.0 mL GOPOD reagent and incubated at 45 °C for 20 min. The absorption value of the blend was determined at 510 nm. RDS (rapidly digestible starch), SDS (slowly digestible starch) and RS (resistant starch) contents were calculated via the following equations:

G_t_ = A_sample_/A_standard_ × 100 (2)

RDS (%) = 0.9 × (G_20_ − G_0_)/TS × 100% (3)

SDS (%) = 0.9 × (G_120_ − G_20_)/TS × 100% (4)

RS (%) = 100 − RDS − SDS(5)

where A_sample_ and A_standard_ represent the absorbance values of samples and glucose standard solutions, respectively; G_t_ stands for glucose content at any time; G_0_, G_20_ and G_120_ are the glucose contents at 0 min, 20 min and 120 min, respectively. TS represented the content of total starch.

### 2.9. Total Plate Count (TPC)

Refer to the method of Zhang et al. [17]. The noodles were blended with 0.85% (*w*/*v*) sterile saline solution and homogenized. A series of 10-fold diluents were prepared for the homogenized samples with the sterile saline. A total volume of 1 mL of the solution was added into the plate count agar, and incubated at 36 °C for 48 ± 2 h. TPC was counted directly through final colony units on the plate. 

### 2.10. Scanning Electron Microscope (SEM)

Refer to the method of Ghoshal et al. [25]. SEM (TM 4000 Plus, Hitachi, ORE, Japan) was used to observe the microstructure of the transverse cross-section of noodles. Prior to being photographed for observation, the noodles were freeze-dried and coated with gold in a sputter coater.

### 2.11. Statistics Analysis

Data were expressed as average ± standard deviation (SD). Figures were drawn using the Origin 2021 software (Origin Lab, Inc., Northampton, MA, USA). Statistical analysis was carried out using SPSS 21.0 software (IBM Inc., Armonk, NY, USA). Different letters indicate significant differences (*p* ≤ 0.05). All experiments were measured at least three times.

## 3. Results

### 3.1. Color Analysis

Color is an important index that affects the sensory qualities of noodles. In general, YANs appear yellow because the alkali causes flavonoids in the flour to produce a color shift [26]. The effect of different amounts of native KGM or CK on the color of the noodles is shown in Table 2. Increasing values of L, a and b meant that the noodles turned bright, red and yellow, respectively. Conversely, decreasing values of L, a and b indicated that the noodles turned dark, green and blue, respectively [27]. There were significant differences in three chromaticity values of the noodles made from native KGM and CK at any point of the same addition. Compared to native KGM, noodles with CK had larger values of L and a, with smaller values of b at the same amount. From the appearance, CK provided the noodles with a higher brightness and a weaker yellow. The color difference (seen in ∆E values) of the noodles occurred regardless of the amounts of KGM or CK powder. It might be from color differences between native KGM powder and CK powder. Furthermore, it was speculated that the two powders could cause differences in the noodles’ gluten network structure, which would consequently affect the reflectivity of light and finally result in color changes [22]. 

### 3.2. Texture Analysis

**Figure 1:**
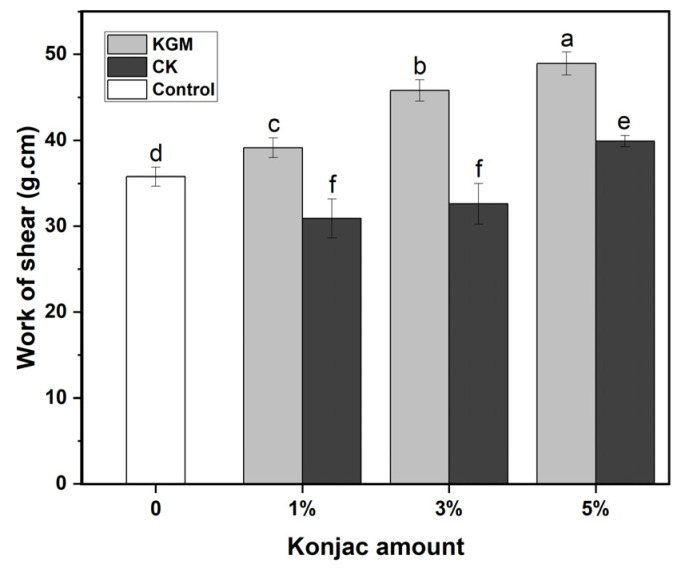
Shear work of yellow alkaline noodles with different levels of native KGM or CK. KGM: konjac glucomannan; CK: chitosan-coated konjac glucomannan. Different letters denote significant difference (*p* ≤ 0.05).

**Figure 2:**
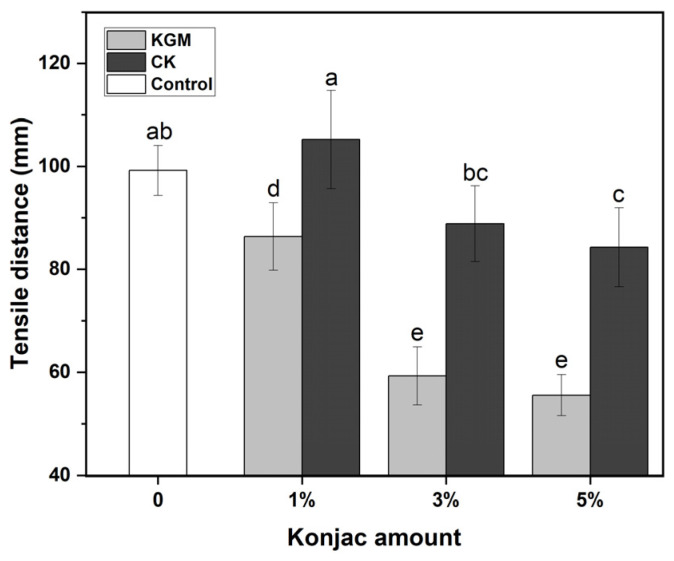
Tensile distance of yellow alkaline noodles with different levels of native KGM or CK. KGM: konjac glucomannan; CK: chitosan-coated konjac glucomannan. Different letters denote significant difference (*p* ≤ 0.05).

The texture properties of the noodles were determined via two methods: shear and tensile. The effects of KGM and CK on the texture of the noodles are presented in Figure 1 and Figure 2. With the increased amounts of KGM or CK, all of the shear work applied to any kind of noodles increased, but the tensile distances of noodles showed a downward trend. When the proportion of konjac powder in flour reached 5%, the shear work required by the KGM noodles and CK noodles were 48.9 g·cm and 39.9 g·cm, respectively, which were higher than that of the control, while the stretching distances of KGM noodles and CK noodles were 55.56 cm and 84.28 cm, respectively, which were lower than that of the control. At the same amount, the shear work of the noodles with KGM was higher than that of CK, but the tensile property of the noodles with KGM was smaller than that of CK. Because the hydrophilicity of KGM and CK was obviously different, a large number of hydroxyl groups existed in the molecular chain of KGM, which had strong hydrophilicity. During the dough production, KGM easily competed with wheat protein for water. As for CK powder, it was obtained by wrapping insoluble chitosan sol in native KGM powder, so the hydrophilicity of CK declined significantly. This meant that the ability of CK to combine with water in the dough was relatively weaker. In brief, compared to CK, KGM could largely prevent wheat protein from binding to water, resulting in damage to the gluten network structure [14,28]. In the preparation of YANs, it was obviously observed that when the mass fraction of KGM reached 5%, undissolved dry powder clumps occurred in the noodles, and the extruded noodles became very dry and crisp. Moreover, hydrated KGM filled the gluten network and increased the firmness of the dough, manifesting as an enhanced shear work [29]. As a spatial barrier, KGM was prone to cause discontinuity in the network structure of gluten, leading to a decline in the ductility or tensile properties of the dough [30].

The occurrence of the above phenomenon might be from the interaction among KGM–gluten protein, KGM–starch, CK–gluten protein and CK–starch. In addition to hydroxyl, the amino group, as a special group on chitosan, also interacted covalently and non-covalently with protein and starch in noodles, thus affecting the structure of the gluten network [17,31]. It was reported that chitosan could improve the gel network strength as well as the ductility of the noodles [25]. The structure change of gluten protein was simply a non-negligible aspect for the textural difference of noodles. 

### 3.3. Water Absorption Analysis

**Figure 3:**
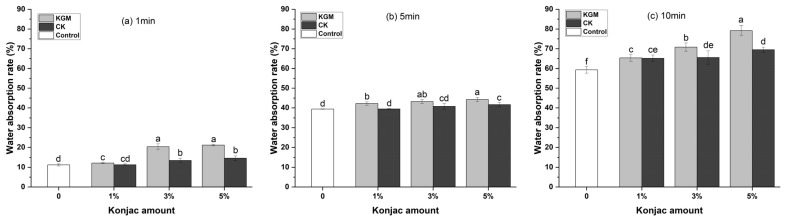
Water absorption rate of yellow alkaline noodles with different levels of native KGM or CK at various time. KGM: konjac glucomannan; CK: chitosan-coated konjac glucomannan. Different letters denote significant difference (*p* ≤ 0.05).

In order to evaluate the cooking quality of noodles, the water absorption rate of YANs was measured by cooking noodles in boiling water for different periods of time [32]. As shown in Figure 3, the water absorption rate of noodles increased with the extension of time. In the cooking process, the hydration of starch and protein occurred, accompanied by the gelatinization of starch and the expansion of the gluten network. When the cooking time was as short as 1 min and the amount of KGM reached 3% and 5%, the water absorption rate of the noodles was about 20%. When the time was extended to 5 min, the noodles appeared in the semi-cooked state, and the water absorption rate of the noodles was around 45%. When further boiled for another 10 min, the noodles were fully cooked and the water absorption rate was close to 80%. Throughout the entire boiling process, the konjac noodles absorbed more water than the control, and the water absorption rate of CK noodles was lower than that of KGM noodles at the same dosage. Especially at higher levels, such as 3% and 5%, the difference between the water absorption rate of CK noodles and KGM noodles seemed to be obvious as KGM is highly hydrophilic and so easily competes for water with wheat protein in dough, resulting in an incompletely hydrated gluten protein. After KGM was coated with chitosan and applied to noodles, the effect of the water absorption rate of the noodles decreased significantly, indicating that chitosan could inhibit the water absorption ability of KGM and slow down the destruction of the gluten protein by KGM to a certain extent. 

### 3.4. Water Distribution Analysis

The water state and water distribution in food were measured via LF-NMR. Changes of water distribution in YANs with different levels of native KGM or CK are shown in Table 3. The water state can be divided into three categories, such as bound water, weakly bound water and free water. They are denoted by T_21_, T_22_ and T_23_, respectively. The smaller the T_2_ value, the more tightly water binds to macromolecules [13]. There were two states of water in YANs, including bound water and weakly bound water. With the increased amount of KGM and CK, T_21_ and T_22_ both showed a downtrend. All T_2_ values of konjac noodles were almost lower than those of the control. It meant that KGM and CK redistributed the water in the noodles, and the water was more tightly locked in the gluten network structure. At the same dosage, the T_2_ values of KGM samples were all smaller than CK samples. Compared to CK, KGM more strongly restricted the movement of water molecules and reduced the relaxation time due to the strong hydrophilicity and high water binding capacity of KGM. The hydroxyl group on the molecular chain reduced the mobility of adsorbed water through proton exchange. 

### 3.5. Starch Digestion Analysis

As seen in Table 4, with the increased amount of KGM or CK, RDS showed a downward trend and SDS showed an upward trend. The RSD content of konjac noodles were obviously lower than that of control, and SDS content and RS content were higher than that of control. This indicated that no matter what form of KGM was added, the digestion rate of starch could be effectively reduced. Glucomannan delayed the starch digestion rate via the strong interaction of KGM–amylose and blocking the binding of amylopectin chains [12].

Moreover, no significant difference occurred in the RS content in noodles as the CK amount changed. However, the RS content of CK noodles was slightly higher than that of KGM noodles at the same addition. It was suggested that a small amount of chitosan distributed in CK might interact with linear macromolecular chains through hydrogen or covalent bonds during heat treatment to form a thin film that covered the surface of starch particles, thus protecting the starch from amylase attacks and increasing the RS content [33]. Alternatively, chitosan might form a complex with starch through interaction, and its structure was not easily hydrolyzed by amylase during digestion [34]. The increase in RS was conducive to the stability of glucose metabolism and the control of diabetes. 

### 3.6. Total Plate Count (TPC) Analysis 

**Figure 4:**
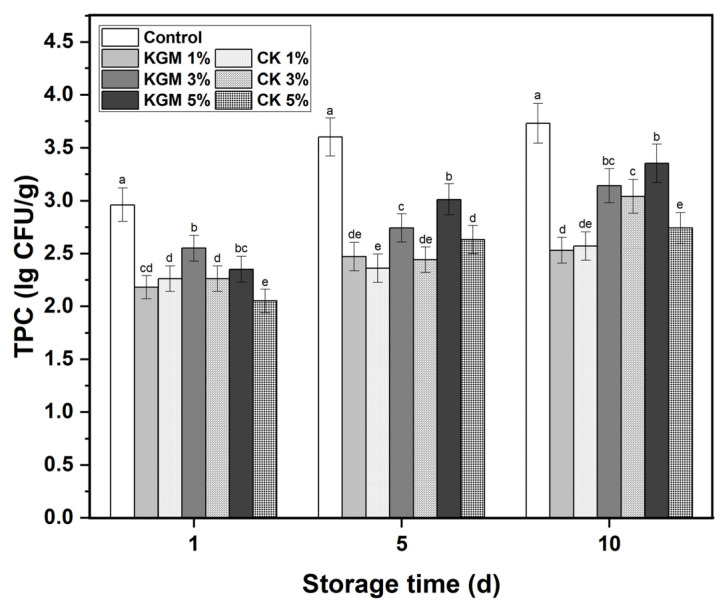
Changes in the total plate count of yellow alkaline noodles with native KGM or CK during storage. KGM: konjac glucomannan; CK: chitosan-coated konjac glucomannan. Different letters denote significant difference (*p* ≤ 0.05).

TPC is an essential indicator to evaluate the shelf life of food that can reflect the microbial growth of food during storage [35]. After fresh and wet YANs were stored at 4 °C for different time periods, the TPC values of noodles with KGM or CK were altered, as illustrated in Figure 4. TPC values increased with time, indicating that the microorganisms in the noodles continued to multiply and grow throughout the storage. The TPC values of control were notably higher than that of konjac noodles during the entire storage. At a lower dosage of 1%, there was no significant difference in TPC value between CK noodles and KGM noodles. However, when the dosage reached 5%, the TPC value of CK noodles was much lower than that of KGM noodles. The phenomenon was observed in the whole storage. It was attributed to the positively charged amino group on the molecular chain of chitosan, which interacted with the cell membrane of the microbial and prevented the transport of molecules inside and outside the cell, making the microbial unable to maintain basic metabolism. It also led to the leakage of cellular components such as microbial proteins, and inhibited the normal growth of microorganisms [16,36]. Therefore, it was concluded that the storage performance of CK noodles was better than that of KGM noodles. The presence of small amounts of chitosan in KGM could help to extend the shelf life of noodles. 

### 3.7. Microstructure Analysis

**Figure 5:**
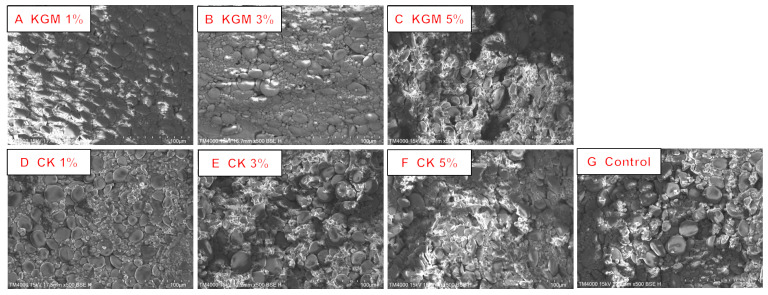
Scanning electron micrographs of cross-sections of yellow alkaline noodles with different levels of native KGM or CK. KGM: konjac glucomannan; CK: chitosan-coated konjac glucomannan.

The internal structure of noodles is closely related to the quality of the noodles [23]. The microstructure and morphology of the noodles added with different amounts of KGM and CK are shown in Figure 5. At the addition of KGM as low as 1%, the noodles presented a compact structure with a relatively flat cross-section, and a large number of starch particles were embedded in the protein network structure. With a greater content of KGM, the cross-section of noodles changed from flat to rough, and the porosity of the cross-section increased. At the highest dose of 5%, it was clearly observed that the protein matrix seemed to be in a torn state with a discontinuous network structure. At this time, the gluten network could no longer wrap the starch particles well, and most of the starch particles were exposed. This was primarily due to the fact that KGM competed to absorb water in the dough, causing the inadequate hydration of wheat protein and blocking the formation of the gluten protein network structure [14,37]. Compared with KGM noodles, CK noodles displayed less flat cross-sections. Except for the starch particles, a large number of small fragments was found in the gluten protein matrix. It was speculated that the fragments were CK powder with poor water solubility that would not only affect the network structure of gluten protein by interaction, but also fill the network structure in a physical barrier. 

## 4. Discussion

As a soluble dietary fiber, KGM has strong hydrophilicity and produces high viscosity. It was found that when the proportion of KGM in flour reached 5%, the dough became dry and rough due to the difficult gluten formation, and the stretching property of the noodles decreased significantly. During the cooking process, the noodles absorbed too much water and became fat, showing an unattractive appearance. This awful phenomenon is attributed to the fact that KGM competed to adsorb the water in the dough, resulting in not enough water participating in the formation of the gluten network structure. 

However, the hydrophilicity of KGM decreased significantly after it was embedded with chitosan sol. When CK powder was added to the noodles, the quality of the noodles changed. Compared with KGM noodles, CK noodles presented a higher tensile property and lower hardness, and the swelling of the noodles was alleviated. It was mainly due to the fact that chitosan reduced the hydrophilicity of KGM, preventing KGM from binding with water in the dough to some extent. This was well confirmed in the experiment of water distribution. It was also speculated that the presence of a small amount of chitosan in noodles altered the gluten network structure through the interaction between the characteristic amino group on a molecular chain with starch or protein in dough. 

It was noteworthy that CK noodles owned other advantages. In the experiment of starch digestion, it was observed that the content of resistant starch in CK noodles increased, which had a positive effect on the regulation of blood glucose. The increased content of resistant starch in noodles once again proved the interaction between chitosan and starch along with the change of starch structure. Furthermore, CK noodles appeared brighter and in a lighter yellow hue—characteristics which are more accepted and preferred by consumers. The appearance discrepancy of the noodles was due in part to color differences between CK powder and KGM powder. Another reason could be that the two different konjac powders brought about different network structures of the gluten, which affected the light reflection and led to color changes in the noodles. Moreover, compared to that of KGM noodles, the TPC value of CK noodles declined during storage. This meant that the shelf life of the noodles was extended with the help of the antibacterial action of chitosan. It was reported that positively charged amino groups from the molecular chain of chitosan could interact with the microbial cell membrane and prevent the transport of molecules inside and outside the cells, so that the microorganisms could not maintain basic metabolism, lead to the leakage of microbial proteins and other cellular components, and thereby inhibit the normal growth of microorganisms. In general, the physicochemical properties and storage properties of CK noodles were superior to those of KGM noodles. The current study provided potential for the application of CK as a new dietary fiber in food processing. It also helped to improve the qualities of traditional flour products. In the future, the digestion process of CK noodles in the stomach and small intestine will be the focus of our study. A relationship between noodle digestion and satiety is expected to be established to provide reference for health food. 

## 5. Conclusions

KGM powder that was coated with chitosan sol could further improve the qualities of noodles. The noodles appeared brighter and in a lighter yellow hue. As for texture, the firmness of the noodles reduced, but the tensile properties rose. In the cooking process, the water absorption of the noodles decreased. In addition, the resistant starch content of the noodles increased. During the storage period, the decrease in TPC in the noodles indicated that the shelf life of the noodles was successfully prolonged. 

## Figures and Tables

**Table 1:** Ingredients for yellow alkaline noodles (g).

Sample	Flour	KGM	CK	Water	Soda	Salt
Control	100.0	0	0	35.0	0.6	0.8
KGM 1%	99.0	1.0	0	35.0	0.6	0.8
KGM 3%	97.0	3.0	0	35.0	0.6	0.8
KGM 5%	95.0	5.0	0	35.0	0.6	0.8
CK 1%	99.0	0	1.0	35.0	0.6	0.8
CK 3%	97.0	0	3.0	35.0	0.6	0.8
CK 5%	95.0	0	5.0	35.0	0.6	0.8

KGM: konjac glucomannan; CK: chitosan-coated konjac glucomannan.

**Table 2:** Chrominance values of yellow alkaline noodles with different levels of native KGM or CK.

Sample	L	a	b	∆E
Control	88.50 ± 0.02 ^a^	−1.40 ± 0.02 ^b^	20.38 ± 0.23 ^b^	
KGM 1%	86.03 ± 0.17 ^f^	−1.41 ± 0.01 ^b^	22.55 ± 0.31 ^a^	3.29 ± 0.17 ^b^
KGM 3%	86.73 ± 0.01 ^e^	−1.50 ± 0.05 ^a^	19.69 ± 0.21 ^c^	1.91 ± 0.21 ^c^
KGM 5%	85.47 ± 0.01 ^g^	−1.53 ± 0.03 ^a^	18.40 ± 0.24 ^d^	3.63 ± 0.02 ^a^
CK 1%	87.85 ± 0.01 ^b^	−1.38 ± 0.03 ^b^	20.19 ± 0.26 ^b^	0.68 ± 0.03 ^f^
CK 3%	87.09 ± 0.11 ^d^	−1.13 ± 0.02 ^c^	20.38 ± 0.33 ^b^	1.45 ± 0.13 ^e^
CK 5%	87.41 ± 0.03 ^c^	−0.93 ± 0.01 ^d^	17.75 ± 0.32 ^e^	2.89 ± 0.09 ^d^

L, lightness; a, redness–greenness; b, yellowness–blueness; ∆E, color difference; KGM, konjac glucomannan; CK, chitosan-coated konjac glucomannan. Different superscript letters denote significant difference (*p* ≤ 0.05).

**Table 3:** Relaxation times (T_21_ and T_22_) of yellow alkaline noodles with different levels of native KGM or CK.

Sample	T_21_/ms	T_22_/ms
Control	2.58 ± 0.00 ^a^	44.49 ± 0.02 ^a^
KGM 1%	1.96 ± 0.00 ^b^	19.34 ± 0.13 ^b^
KGM 3%	1.48 ± 0.01 ^c^	14.65 ± 0.09 ^c^
KGM 5%	1.29 ± 0.00 ^d^	11.90 ± 0.40 ^d^
CK 1%	3.18 ± 0.01 ^e^	31.44 ± 0.23 ^e^
CK 3%	2.25 ± 0.00 ^f^	27.36 ± 1.00 ^f^
CK 5%	1.59 ± 0.00 ^g^	16.83 ± 0.10 ^g^

T_21_ and T_22_ represent the relaxation time of bound water and weakly bound water, respectively. KGM: konjac glucomannan; CK: chitosan-coated konjac glucomannan. Different superscript letters denote significant difference (*p* ≤ 0.05).

**Table 4:** Starch digestion indexes of yellow alkaline noodles with different levels of native KGM or CK.

Sample	RDS (%)	SDS (%)	RS (%)
Control	73.64 ± 0.10 ^a^	9.55 ± 0.10 ^g^	16.82 ± 0.11 ^c^
KGM 1%	64.98 ± 0.12 ^d^	16.96 ± 0.10 ^d^	18.06 ± 0.16 ^a^
KGM 3%	63.70 ± 0.11 ^f^	18.63 ± 0.09 ^b^	17.67 ± 0.14 ^b^
KGM 5%	62.66 ± 0.11 ^g^	19.76 ± 0.11 ^a^	17.58 ± 0.16 ^b^
CK 1%	69.33 ± 0.12 ^b^	12.46 ± 0.09 ^f^	18.21 ± 0.15 ^a^
CK 3%	66.06 ± 0.09 ^c^	15.86 ± 0.08 ^e^	18.08 ± 0.12 ^a^
CK 5%	64.08 ± 0.12 ^e^	17.95 ± 0.08 ^c^	17.97 ± 0.14 ^a^

RDS: rapidly digestible starch; SDS: slowly digestible starch; RS: resistant starch; KGM: konjac glucomannan; CK: chitosan-coated konjac glucomannan. Different superscript letters denote significant difference (*p* ≤ 0.05).

## Data Availability

No new data were created or analyzed in this study. Data sharing is not applicable to this article.
